# P-30. Herpes Zoster Burden among US Adults with Immunocompromising or Comorbid Conditions: A Systematic Literature Review

**DOI:** 10.1093/ofid/ofae631.237

**Published:** 2025-01-29

**Authors:** Justin Gatwood, Sophie Dodman, Aiswarya Shree, Bhanu Inuganti, Carol A Forbes, Nikita Stempniewicz

**Affiliations:** GSK, Philadelphia, Pennsylvania; Evidera, London, England, United Kingdom; Evidera, London, England, United Kingdom; Evidera, London, England, United Kingdom; Evidera, London, England, United Kingdom; GSK, Philadelphia, Pennsylvania

## Abstract

**Background:**

Prior to and since the availability of herpes zoster (HZ) vaccines, the burden of HZ has been summarized across select US patient populations, including those with comorbidities, autoimmune disorders (AID), or immunocompromising (IC) conditions. However, these studies tended to focus on disease incidence, lacked comparisons across multiple conditions, and generally did not include healthcare utilization and cost outcomes associated with HZ. Consequently, this study sought to summarize HZ burden in terms of epidemiology, health outcomes, health care resource utilization and costs, and humanistic impact among those with AID, IC conditions, or comorbidities in the US.

**Table.**
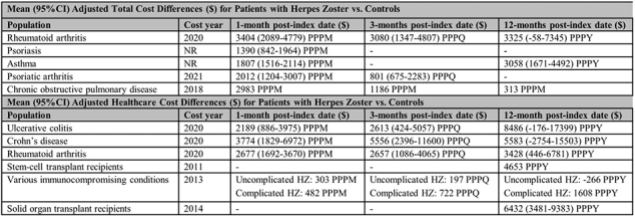
Incremental Health Care Costs for Patients with Herpes Zoster among Adults with AID, IC, or Common Comorbid Conditions AID = autoimmune disorders; CI = confidence interval;IC = immunocompromising , USD ($) = United States dollar; NR = not reported; PPPM = per patient per month; PPPQ = per patient per quarter (3-month period); PPPY = per patient per year

**Methods:**

This systematic literature review involved 3 separate searches to identify studies published since 2012 reporting on the epidemiology, economic burden, and humanistic burden of HZ in US adults with AID, IC conditions, or common comorbidities. Methods followed those recommended by the Cochrane Collaboration and involved searches within Embase, MEDLINE, proceedings from relevant conferences (2021 to 2023), and the CDC website. Study selection was carried out by 2 independent reviewers and data were extracted from the included studies by 1 reviewer and validated by a second reviewer.

**Figure 1.**
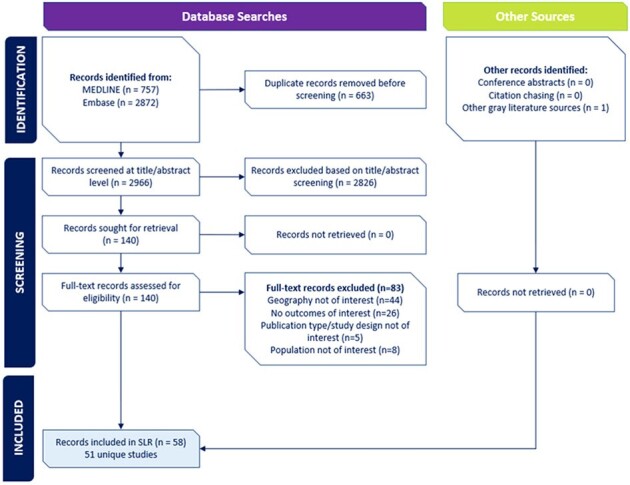
PRISMA Flow Diagram: Epidemiological Study Selection SLR = systematic literature review

**Results:**

The final review included 51 epidemiological studies (Figure 1), 18 economic analyses (Figure 2), and no publications on humanistic burden. Extensive evidence on HZ incidence was observed across 26 individual AID, IC, or common conditions, with the highest rates among those with hematological malignancies or stem cell transplant recipients. Limited evidence on HZ-related mortality or the prevalence of other HZ-related complications was observed in AID and IC populations. Economic burden of HZ varied considerably by underlying condition(s) (Table) with the majority of incremental costs and resource utilization observed within the first month following HZ diagnosis, primarily driven by inpatient care.

**Figure 2.**
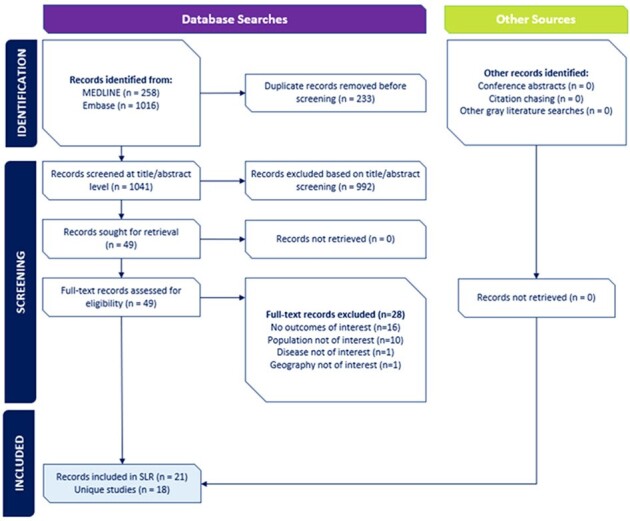
PRISMA Diagram: Economic Study Selection SLR = systematic literature review

**Conclusion:**

HZ burden is considerable among adults with AID, IC, and/or common comorbid conditions. The observed risk for and burden of HZ reinforces the need to expand vaccination coverage among adults in these patient populations.

FUNDING: GSK (VEO-000677)

**Disclosures:**

**Justin Gatwood, PhD, MPH**, AstraZeneca: Grant/Research Support|Genentech: Advisor/Consultant|GSK: Employment|GSK: Stocks/Bonds (Public Company)|Merck & Co.: Advisor/Consultant|Merck & Co.: Grant/Research Support **Sophie Dodman, BSc**, Evidera (part of Thermo Fisher): Employee **Aiswarya Shree, MSc**, Evidera (part of Thermo Fisher): Employee **Bhanu Inuganti, MSc**, Evidera (part of Thermo Fisher): Employee **Carol A. Forbes, PhD**, Evidera (part of Thermo Fisher): Employee|Evidera (part of Thermo Fisher): Stocks/Bonds (Public Company) **Nikita Stempniewicz, MSc**, GSK: Employment|GSK: Stocks/Bonds (Public Company)

